# Survivin regulates the expression of VEGF-C in lymphatic metastasis of breast cancer

**DOI:** 10.1186/1746-1596-7-52

**Published:** 2012-05-18

**Authors:** Xiaopeng Cai, Shuai Ma, Ming Gu, Cong Zu, Wenzhi Qu, Xinyu Zheng

**Affiliations:** 1 Department of Breast Surgery, First Affiliated Hospital, China Medical University, No. 155 North Nanjing Street, Shenyang, Liaoning Province, 110001, China; 2 Lab 1, Cancer Institute, China Medical University, Shenyang, Liaoning Province, 110001, China; 3 Department of Breast Surgery, Fourth Affiliated Hospital, China Medical University, Shenyang, Liaoning Province, 110001, China

**Keywords:** Survivin, VEGF-C, Breast cancer, Lymphatic metastasis

## Abstract

**Background:**

As a known regulator of apoptosis, survivin has positive relationship with lymphatic metastasis in breast cancer. This study aims to detect the difference in expression between survivin and vascular endothelial growth factor-C (VEGF-C) in treated breast cancer cells and tissues, and to analyze the correlation among survivin, VEGF-C and lymphatic metastasis.

**Methods:**

Plasmid with survivin and VEGF-C shRNA and lentivirus with survivin gene were constructed and transfected into breast cancer cell ZR-75-30. Then the expressions of the two genes were examined using western blot analysis and real-time PCR. The change of invasiveness of breast cancer cells was assessed using matrigel invasion assay. Using immunohistochemistry, the expression of survivin and VEGF-C were analyzed in 108 clinical breast cancer cases with breast cancer tissue and lymph node.

**Results:**

Survivin regulated the expression of VEGF-C at both protein and mRNA levels in breast cancer cells. Immunohistochemical analysis showed that the level of VEGF-C expression was significantly related with that of survivin in breast cancer tissues (*p*<0.05). VEGF-C was found to participate in the process of breast cancer cells invasion mediated by survivin. The co-expression of the two and the single expression of any one took significant difference in positive lymph node (*p*<0.05).

**Conclusions:**

Survivin takes an important part in regulating the expression of VEGF-C. VEGF-C could influence the invasive ability mediated by survivin. The co-expression of survivin and VEGF-C is more statistically significant to assess lymphatic metastasis in breast cancer.

**Virtual slides:**

The virtual slide(s) for this article can be found here: http://www.diagnosticpathology.diagnomx.eu/vs/9193530897100952

## Background

Breast cancer is the most frequently diagnosed cancer and the leading cause of cancer death in females, accounting for 23% (1.38 million) of the total new cancer cases and 14% (458,400) of the total cancer deaths in 2008 worldwide [1]. More than half of breast cancers are associated with axillary nodal involvement. Metastasis and recurrence severely affect the quality and length of lives of breast cancer patients. In the NCCN clinical practice guidelines of breast cancer, lymphatic metastasis is considered the main criteria for clinical prognosis and target of systemic treatment. Axillary lymph node status is the most important prognostic factor in breast cancer, and prognosis declines with increasing number of tumor-positive lymph nodes [2].

Lymphatic metastasis is one of the most important pathways of breast cancer systemic metastasis, and is closely related to prognosis and therapy plans for breast cancer patients. Many pathways have been suggested to be involved in the process of breast cancer lymphatic metastasis. And some markers, such as EGFR [3] and BCRP [4], could be detected as the predictable target of breast cancer lymphatic metastasis. Some researches have indicated that survivin and vascular endothelial growth factor-C (VEGF-C) may take part in the course of lymphatic metastasis of breast cancer.

Survivin is one of the inhibitors of apoptosis protein (IAP). It regulates two important cellular processes including inhibition of cell apoptosis and promoting cell proliferation [5]. High levels of survivin mediate resistance of cancer cells to a series of anti-cancer drugs [6], and it has been shown that up-regulation of survivin confers resistance of cancer cells to radiotherapy [7,8]. Several researches in breast cancer, gastric cancer, oral squamous cell carcinoma and colorectal cancer have shown that the expression of survivin is significantly related to lymphatic metastasis, and that survivin is the prognostic marker for these cancers [9-13]. However, how survivin controls lymphatic metastasis remains elusive.

VEGF-C, also called lymphatic vessel growth factor, is a lymphatic endothelial cell-stimulating factor. VEGF-C affects blood vessel and lymphatic vessel through vascular endothelial growth factor receptor-2 (VEGFR-2) and vascular endothelial growth factor receptor-3 (VEGFR-3), respectively, to improve tumor growth and metastasis [14]. High levels of VEGF-C have been detected inside or around tumors lymphatic vessels, by which lymphatic and even distal metastasis are promoted [15]. High levels of VEGF-C also lead to lymphatic vessel formation in lymph nodes [16]. Many researches have also demonstrated that VEGF-C is involved in lymphatic invasion in esophageal cancer, breast cancer, non-small cell lung cancer, and colorectal cancer [17-20].

It has been suggested that both survivin and VEGF-C play important roles in tumor lymphatic metastasis; however, studies on the relationship between the two are scarce. In this study, we discuss the relationship between survivin and VEGF-C in breast cancer and the pathway by which survivin may affect breast cancer lymphatic metastasis.

## Material and methods

### Clinical samples

A total of 108 breast cancer patients aged 32 to 63 with a mean age of 48 were involved in this study. All patients were examined and monitored from 2009-2011 in the First Affiliated Hospital of China Medical University in Shenyang, Liaoning province, China. Breast cancer was diagnosed and classified into various stages according to the International Union Against Cancer (UICC) and the TNM classification system published by the American Joint Committee on Cancer (AJCC). Clinical information obtained from the records and the histopathology reports included age, first diagnosis, tumor size and grade, oestrogen-receptor (ER) and progesterone-receptor (PR) status, and lymph nodal involvement.

### Cell culture

Human breast cancer cell line ZR-75-30 was preserved from Cell Resoure Center of Shanghai Life Science Research Institute, Chinese Academy of Sciences. And it was cultured in RPM1640 medium (Invitrogen Corporation, Carlsbad, California, USA) supplemented with 50 μg/ml penicillin, 50 μg/ml streptomycin and 10% fetal bovine serum at 37°C in a 5% CO_2_ environment. Long phase cells were collected after trypsin digestion by centrifugation for 5 minute at 1,000 rpm, re-suspended in phosphate buffered saline (PBS), and counted using a haemocytometer.

### Construction and transfection of plasmid-ShRNA and lentivirus

Plasmids with survivin-shRNA and VEGF-C-shRNA were constructed by Shanghai GennePharma Co., Ltd, and lentivirus with survivin gene was constructed by Shanghai GeneChem Co., Ltd. Plasmid-survivin-shRNA and plasmid-VEGF-C-shRNA, 10 μg shRNA and 25 μl Lipofectamine 2000 (Invitrogen Corporation, Carlsbad, California, USA) were mixed in 1350 μl 1640 medium without FBS and transfected into brest cancer cells. The mixture were added into 25-cm^2^ culture flask that was previously plated with 1 × 10^6^ ZR-75-30 breast cancer cells. Culture medium was replaced with complete 1640 medium once six hours post inoculation and cells were collected after another 28 hours. Protein and RNA were extracted for western blot and real-time PCR analysis, respectively.

Lentivirus with survivin gene was transfected into cells at an MOI of 20 and 0.75 μl polyprene were added into each well of the 6-well plate containing 2 × 10^5^ ZR-75-30 cells. Medium was replaced after 8 hours, and then cells were cultured at 37°C in a 5% CO_2_ environment.

### Western blot analysis

Proteins were separated by SDS-PAGE, transferred onto nitrocellulose membranes, and detected using relevant primary antibody and appropriate secondary antibody. Primary antibodies included: anti-survivin (mouse monoclonal, Santa Cruz, CA, USA), anti-VEGF-C (rabbit polyclonal, ABGENT, San Diego, CA, USA) and anti-β-actin (mouse monoclonal, Santa Cruz, CA, USA).

### RNA isolation and Real-time polymerase chain reaction

Total RNA was extracted using the guanidinium thiocyanate-phenol-chloroform method. RNA yield and purity were determined photometrically (BioPhotometer, Eppendorf, Germany). Reverse transcription was performed. Survivin and VEGF-C were amplified using real time PCR. A total of 10 ng of reverse-transcribed total RNA was used as the template, and PCR reaction contained 20 pmol/ml of each sense and antisense primer (Table 1) and SYBR Premix Ex Taq II (TaKaRa, Dalian, Shenyang, China) in a final volume of 20 μl. An ABI PRISM 7700 Sequence Detection System Instrument (Applied Biosystems) was used for the amplification. Cycling conditions consisted of an initial denaturation step at 95°C for 10 min as a ‘hot start’, followed by 40 cycles of 95°C for 15 s, annealing temperature for 30 s, 72°C for 30 s, and a final extension at 72°C for 10 min. GAPDH was used in each experiment as an endogenous control. Relative quantification for a gene was expressed as fold changes over the control group. Fold changes were calculated using the 2^- ΔΔCt^ method.

**Table 1:** Primers used for real-time PCR

**Primer**		**5′-3′ sequence**	**Size of PCR (bp)**
**Survivin**	**forward**	TCATAGAGCTGCAGGGTGGATTGT	114
	**reverse**	AGTAGGGTCCACAGCAGTGTTTGA	
**VEGF-C**	**forward**	AACCTCCATGTGTGTCCGTC	156
	**reverse**	TGGCAAAACTGATTGTTACTGG	
**GAPDH**	**forward**	ACAGTCCATGCCATCACTGCC	266
	**reverse**	GCCTGCTTCACCACCTTCTTG	

### Matrigel invasion assay

The migration capacity of breast cancer cells was determined in vitro using Transwell Chambers (Corning, NY, USA) in which the two chambers were separated with matrigel coated polycarbonate membrane (6.5-mm diameter inserts, 8 μm pore size). Breast cancer cells (5 × 10^5^/ml serum-free medium) were placed in the upper chamber (200 μl). The lower chamber contained medium alone (500 μl). Chambers were assembled and kept in an incubator for 24 hours. At the desired time point, cells from the upper surface of the membrane were removed with gentle swabbing and the migrant cells on the lower surface of the membrane were fixed by methanol and stained with crystal violet. Then membranes were washed with PBS and mounted onto slide glasses. The membranes were visualized microscopically (Olympus BX41) and cellular migration per sample was determined by counting the number of stained cells in at least four to five randomly selected fields. Data are presented as mean number of the migrating cells ± SD per microscopic field per sample. Each cell migration experiment was repeated at least three times.

### Immunohistochemical analysis

Immunohistochemistry was carried out on paraffin-embedded tumor specimens fixed in 4% buffered formalin. Four-micrometer-thick histological slides were de-paraffinized in xylol and heated in 0.01 M citrate buffer for 25 min in a microwave oven. After cooled for 20 min and washed in PBS, endogenous peroxidase was detected by incubating samples with PBS containing 10% normal goat serum for 30 min. Then the sections were incubated with each primary antibody overnight at 4°C. The primary antibodies were anti-survivin (mouse monoclonal, Santa Cruz, CA., USA, 1:10) and anti-VEGF-C (rabbit polyclonal, Abcam Inc, Cambridge, MA, UK, 1:200). A further wash in PBS was followed by treatment with peroxidase-labeled polymer conjugated to goat anti-mouse or anti-rabbit immunogloblins (Envison + kit; Dako, Glostrup, Denmark) as the secondary antibody for 10 min at room temperature. The staining was visualized with diaminobenzidine (DAB), followed by counterstaining with hematoxylin. For a negative control, PBS was substituted for the primary antibody.

The degree of immunohistochemical staining was recorded on a scale of 0–3 according to the percentages of staining and distributions within the cytoplasm. Tumors were scored on a four-tier system: less than 10% of cancer cells staining was designated negative (as degree 0), 10–20% positive staining was scored as degree 1+, 21–50% positive staining was scored as degree 2+, and 51–100% positive staining was scored as degree 3+. We checked at least five 200 × visual fields for one glass slide under microscope, counted the percentage of positive cells in every fields and got the mean percentage of positive cells in a glass slide. At last, we got the score of the glass slide on the base of percentage.

### Statistical analysis

Data was expressed as the means of at least three different experiments ± SD. The results were analyzed by chi-square test and Spearman analysis. *p*<0.05 was considered statistically significant.

## Results

### Transfection of plasmid and lentivirus

Plasmids with survivn shRNA and VEGF-C shRNA contained green fluorescent protein (GFP) were transfected into cells using the method mentioned above. The transfection efficiency was about 50%–70% after 24 hours.

Lentivirus-survivin with GFP was transfected into cells using the method mentioned above. The transfection efficiency was nearly 100%.

### Expressions of VEGF-C and survivin were positively correlated

**Figure 1:**
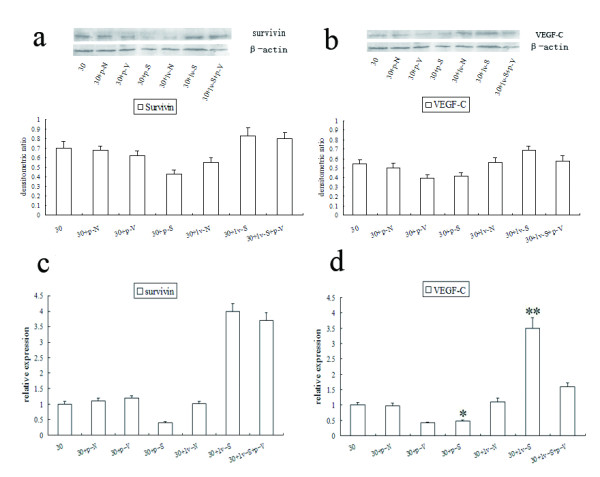
**Expression of survivin and VEGF-C in different processed cells at protein and mRNA level.a** and **b**: western blot analysis for protein expression; **c** and **d**: real-time PCR for mRNA expresison. * means statistically significant decrease comparing to 30, and ** means statistically significant increase comparing to 30 (both *p*<0.05). (30 = ZR-75-30 breast cancer cell line; p-N = plasmid with negative control; lv-N = lentivirus with negative control; p-S = survivin shRNA; p-V = VEGF-C shRNA; lv-S = lentivirus with survivin gene).

In ZR-75-30 cells, when survivin was down-regulated, VEGF-C was down-regulated with western blot method. When survivin was up-regulated, VEGF-C had a higher expression level than that in normal cells. In ZR-75-30 cells over-expressing survivin, VEGF-C expression level also decreased when survivin was down-regulated. Real-time PCR showed that the level of VEGF-C mRNA positively correlated with the level of survivin mRNA (Figure 1).

### Survivin changed invasive ability of cells through VEGF-C

**Figure 2:**
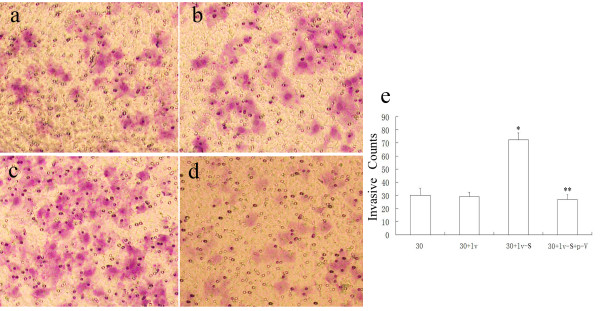
**Matrigel invasion assay from different processed cells.a**,**b**,**c** and **d** show the invasive cells of group 30, group 30 + lv, group 30 + lv-S and group 30 + lv-S + p-V under a microscope(200×). **e** shows the mean number counted from above visual field. * means statistically significant different between 30 + lv-S and ZR-75-30 cells. ** means significant different between 30 + lv-S and 30 + lv-S + p-V(both *p*<0.05).

In the matrigel invasion assay, breast cancer cells with high levels of survivin were more invasive than those with low levels of survivin, which suggested that survivin played an important role in tumor cells migration. However, down-regulated VEGF-C in these cells significantly reduced the number of breast cancer cells that could migrate through the polycarbonate membrane (Figure 2).

### Survivin and VEGF-C expression in tumor tissue and lymph node

**Figure 3:**
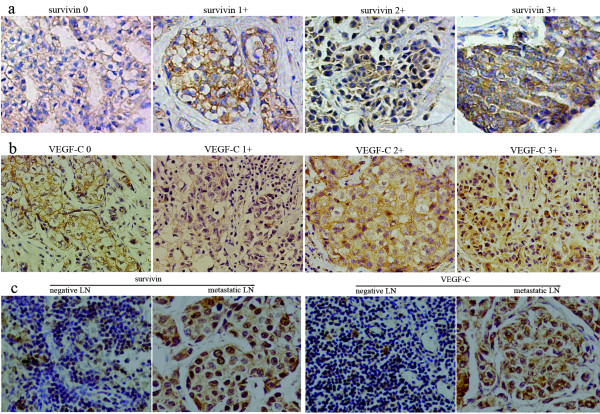
**Results of immunohistochemical staining of breast cancer tissue and lymph node.a** shows survivin expression in the order of degree 0-3+ in breast cancer tissue; **b** shows VEGF-C expression in the order of degree 0-3+ in breast cancer tissue; **c** shows survivin and VEGF-C expressions in negative and metastatic lymph node respectively(200×).

Survivin and VEGF-C mainly localized in the cytoplasm, but could also be detected in the nuclei with nuclear-specific dye. Survivin was expressed in the breast cancer tissue of 83.3% of the patients, among which degree 1-3+ were expressed at 26.7%, 50.0%, 23.3%. In the same group of patients, VEGF-C was expressed at 77.8% and degree 1-3+ were 28.6%, 53.6%, 17.9%. In the lymph node tissue, both survivin and VEGF-C were expressed at higher levels in positive LN than in negative LN (Figure 3).

### Pathological analysis of patients

Survivin and VEGF-C were expressed at higher levels in patients with lymphatic metastasis, and stage III, IV breast cancers (Table 2). The expression levels were also statistically different in tumors of different sizes. However, there were no differences in the expression of the two genes in patients with different age, histological grade, pathological style, or ER/PR status. The results showed that higher expression was detected in C-erBb-2 positive but not in C-erBb-2 negative patients.

**Table 2:** Association of survivin and VEGF-C protein expression with clinicopathological features in breast cancer patients

**Variable**	**n**	**Survivin-**	**Survivin+**	**χ**^**2**^	***p***	**VEGF-C -**	**VEGF-C +**	**χ**^**2**^	***p***
**Age**				0	1			0.965	0.326
**≤45 years**	36	6	30			6	30		
**>45 years**	72	12	60			18	54		
**Tumor size**				9.305	0.01			4.102	0.129
**T1**	39	12	27			12	27		
**T2**	60	6	54			9	51		
**T3**	9	0	9			3	6		
**Histological grade**				0.873	0.350			0.175	0.675
**I + II**	99	15	84			21	78		
**III**	9	3	6			3	6		
**Pathological style**				0.169	0.681			0	1
**ISC**	12	3	9			3	9		
**IC**	96	15	81			21	75		
**Lymph node metastasis**				16.283	0.000			18.544	0.000
**Negative**	30	12	18			15	15		
**Positive**	78	6	72			9	69		
**C-erBb-2**				8.269	0.004			4.354	0.037
**positive(3 + )**	12	6	6			6	6		
**negative(0-2 + )**	96	12	84			18	78		
**ER**				0.617	0.432			0.882	0.348
**positive**	63	9	54			12	51		
**negative**	45	9	36			12	33		
**PR**				0	1			1.929	0.165
**positive**	54	9	45			15	39		
**negative**	54	9	45			9	45		
**TNM stage**				10.800	0.001			6.027	0.014
**I, II**	72	18	54			21	51		
**III, IV**	36	0	36			3	33		

ISC, in situ carcinoma; IC, invasive carcinoma.

There was significant difference between the levels of VEGF-C and survivin expression (*p*<0.05) (Table 3). When the level of survivin expression increased from degree 0 to 3, the level of VEGF-C expression increased correspondingly. And the Pearson coefficient of contingency C = 0.514, which means an actual expression relationship between VEGF-C and survivin.

**Table 3:** Relationship of expression extent between surivivin and VEGF-C

	**VEGF-C 0-1+**	**VEGF-C 2+**	**VEGF-C 3+**	**χ**^**2**^	***p***
**Survvin 0-1+**	30	9	3	38.769	0.000
** Survivin 2+**	12	30	3		
** Survivin 3+**	6	6	9		

In the patients with co-expression of survivin and VEGF-C, the lymph node positive rate is higher than the patients with the single or none expression of survivin and VEGF-C (*p*<0.05) (Table 4). This result indicated that survivin co-operated with VEGF-C in lymphatic metastasis.

**Table 4:** Relationship of co-expression of survivin and VEGF-C with lymph node involvement

	**Survivin(−/+)/VEGF-C(−/+)**	**Survivin(+)&VEGF-C(+)**	**χ**^**2**^	***p***
**LN-**	12	18	4.985	0.026
**LN+**	15	63		

## Discussion

The expression of survivin is high during fetal development but low in healthy adult tissues. However, in most malignant tumors, the expression of survivin increases. As a result, survivin has been considered a potential tumor marker and an important therapeutic target [5]. Studies show that the expression of survivin in many kinds of tumors correlates with lymphatic metastasis. Our results suggest that survivin may influence breast cancer lymphatic metastasis and distal invasion through VEGF-C.

Researches on different tumors have indicated that the up-regulation of VEGF-C promotes tumor lymphatic vessel formation and increases lymph node metastasis. VEGF-C could form lots of lymphatic vessel inside or around the tumor, by which promoting tumor metastasis to lymph node and even distal organs [15]. Then it could make a good condition for advanced metastasis and diffusion.

We showed that the protein and mRNA expression of VEGF-C are controlled by survivin. So, there must be a notal point by which the expression of VEGF-C could be regulated by survivin. Cox-2 activation is highly correlated with VEGF-C expression [21], and through its downstream molecules, cox-2 is able to up-regulate the expression of VEGF-C in cancer cells [22,23]. As an important regulator of apoptosis, cox-2 is usually over-expressed with survivin in hepatocellular carcinoma, surperficial urothelial carcinoma and endometrial carcinoma [24-26]. It is possible that cox-2 may be the notal point to link survivin and VEGF-C expressions.

It has been suggested that the interaction of XIAP and survivin promotes the invasion of tumor cells and enhances the metastatic spread in vivo [27]. Khan et al. have shown that synthetic survivin enhances the proliferation, drug resistance, and cellular invasion of tumor cells [28]. It has been suggested that VEGF-C could promote the VEGFR3 positive tumor cells invade to lymphatic vessels through autocrine and CCR-7 dependent paracrine mechanism [29]. VEGF-C was also proven to control tumor cells growth and invasion by atuocrine mechanism in the carcinoma of gallbladder [30]. In accordance with this result, our results show that the invasiveness of breast cancer cell increases when survivin is over-expressed, and significantly decreases when survivn is over-expressed while VEGF-C is down-regulated. So VEGF-C may play an important role in enhancing the invasiveness in tumor cells caused by survivin. On the other hand, in positive lymph node, survivin and VEGF-C both express at high levels, which may indicate that both of them play important roles in lymphatic metastasis and invasion in breast cancer.

Several researches found that survivin and VEGF-C took high level expression in the breast cancer, also had positive relationship with positive lymphatic metastasis respectively [9,31,32]. And not only in the primary breast tumor, survivin was also found high level expression in circulating tumor cells in peripheral blood through RT-PCR ELISA method [33]. It suggested that the tumor cells with survivin high expression showed great invasive and metastatic ability. In our study, it has been proven that survivin and VEGF-C both are closely related with lymphatic metastasis in different tumors. We show in our study that survivin and VEGF-C expression are positively correlated, and the co-expression of the two is also positively correlated with positive lymph node. This result supports the conclusion based on previous finding that changes in survivin expression induce the changes in VEGF-C expression.

## Conclusion

As a conclusion, survivin is related with breast cancer lymphatic metastasis. It gets correlation with the rate of lymph node metastasis and the invasive ability of breast cancer cells. VEGF-C may play an important role in these processes. Survivin can influenced the expression of VEGF-C to reduce breast cancer lymphatic metastasis and invasion, which helps to decrease death risk of breast cancer. Meanwhile, survivin and VEGF-C can be used simultaneously as important markers to access lymphatic metastasis and distal invasion of breast cancer.

## Competing interests

All authors declare no competing interests.

## Authors’ contributions

XC and SM carried out the design and western blot of this research, MG and CZ participated in real-time PCR and immunohistochemistry of this research, WQ performed the Statistical Analysis, XZ was the director of this research and helped to draft the manuscript. All authors read and approved the final manuscript.
